# Highly Thermally Conductive PDMS/h-BN Composites Enabled by Aspect-Ratio-Driven Alignment

**DOI:** 10.3390/polym18040539

**Published:** 2026-02-22

**Authors:** Mi-Ri An, Ji-Yoon Ahn, Eun-Taek Hor, Sung-Hoon Park

**Affiliations:** Department of Mechanical Engineering, Soongsil University, 369 Sangdo-ro, Dongjak-Gu, Seoul 06978, Republic of Korea; anmiri0622@gmail.com (M.-R.A.); a024679@naver.com (J.-Y.A.); we4245@icloud.com (E.-T.H.)

**Keywords:** thermal management materials, boron nitride, alignment, polymer composite, aspect ratio

## Abstract

Shear-induced alignment of hexagonal boron nitride (h-BN) platelets offers a scalable route to high-performance, electrically insulating thermal management materials, yet the role of filler geometry under practical shear processing remains unclear. Here, we examine how platelet aspect ratio governs alignment and heat transport in PDMS/h-BN composites processed by sequential roll-gap controlled two-roll milling. Using a geometric moment-arm perspective, we relate the platelet effective radius to the shear-driven rotational driving moment. High-aspect-ratio platelets (L-BN) exhibit more stable flow-parallel alignment than small platelets (S-BN), forming a better-connected conductive network. At 175 wt% loading, the aligned L-BN composite achieves 10.3 W m^−1^ K^−1^ (94% higher than its random counterpart) and outperforms the S-BN system while also improving stiffness and device-relevant heat dissipation. These results identify aspect ratio as an alignment-enabling design criterion for scalable thermal management.

## 1. Introduction

Heat accumulation is widely recognized as a major cause of performance degradation and failure in electronic systems [1,2]. With the relentless trend toward miniaturization and higher integration density, heat generation per unit volume has rapidly increased within confined spaces, exacerbating thermal buildup due to insufficient heat dissipation. The resulting localized temperature rise and accelerated thermal stress are key factors that compromise long-term reliability and service lifetime. Therefore, developing high-performance thermal management materials capable of maximizing heat dissipation under high-heat-flux operating conditions is essential [3,4,5,6].

Polymer-based thermal management materials are widely utilized in electronic packaging because they offer excellent flexibility [7], electrical insulation [8], and cost-effective, large-area processability [9,10]. Among them, polydimethylsiloxane (PDMS) is a promising silicone-based matrix due to its superior processability and mechanical compliance. However, its intrinsically low thermal conductivity (~0.2 W m^−1^ K^−1^) is insufficient to meet the thermal requirements of high-power devices [11,12]. To overcome this limitation, composite strategies that incorporate thermally conductive fillers into the PDMS matrix are required to construct efficient thermal pathways within the composite [13,14,15].

A broad range of thermally conductive fillers, including metals, carbon-based materials, and ceramics, have been investigated [8,16,17,18,19,20]. Nevertheless, metallic and carbonaceous fillers often exhibit high electrical conductivity, limiting their applicability in packaging sectors where electrical insulation is mandatory [3,21]. In contrast, hexagonal boron nitride (h-BN), a representative ceramic filler, has attracted considerable attention as a next-generation thermal filler [22] because it simultaneously provides high thermal conductivity, excellent electrical insulation, and outstanding thermal/chemical stability [23]. However, h-BN possesses strong thermal anisotropy arising from its layered crystal structure (in-plane ~300 W m^−1^ K^−1^ versus through-plane ~30 W m^−1^ K^−1^) [24,25]. When h-BN platelets are randomly dispersed in a polymer matrix, filler–filler junctions tend to be discontinuous, diminishing the efficiency of the thermal conduction network [26]. In particular, random dispersion often promotes edge-to-edge contacts rather than parallel overlapping of platelets, hindering the full exploitation of the intrinsic thermal conductivity of h-BN. Accordingly, controlling filler orientation through an alignment strategy is crucial to form continuous, low-tortuosity heat conduction pathways along the dominant heat transport direction [27]. Although electromagnetic-field-assisted alignment [28,29,30] and freeze-casting [31] have been proposed to orient h-BN, these approaches typically involve high process complexity and face limitations in scale-up for continuous, large-area manufacturing. By contrast, mechanical alignment using a shear field offers practical advantages due to its process simplicity and compatibility with continuous production. In particular, two-roll milling can impose strong shear deformation on highly viscous pastes, serving as an industrially viable method to effectively induce filler orientation [32].

**Figure 1:**
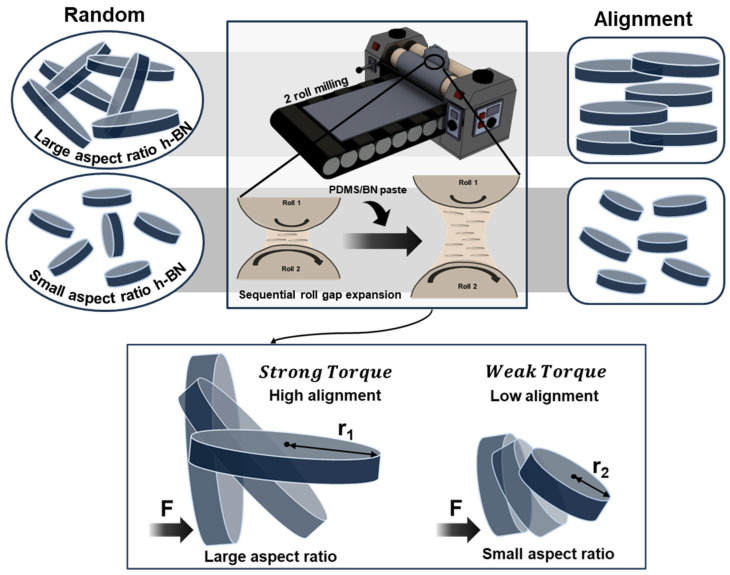
Schematic of aspect-ratio-dependent shear alignment of h-BN in PDMS/BN paste using two-roll milling.

Importantly, however, the efficiency of shear-induced alignment is not determined solely by the magnitude of the applied shear but can be strongly influenced by the filler geometry, especially the aspect ratio [33,34]. According to Jeffery-type rotational dynamics, low-aspect-ratio particles frequently undergo tilting or tumbling under shear flow, making it difficult to maintain a stable, parallel orientation. In contrast, high-aspect-ratio platelets tend to exhibit more stable alignment with respect to the flow direction [35]. Furthermore, as schematically illustrated in Figure 1, shear-driven rotational reorientation can also be interpreted from the perspective of a geometric moment arm [36,37]. The effective platelet radius (*r*) functions as a moment arm for the hydrodynamic torque acting on the particle in the shear field, implying that a larger *r* can generate a larger driving moment for reorientation under identical shear conditions.

While prior studies have largely focused on demonstrating the overall effectiveness of shear processing, this study places the aspect ratio of h-BN at the forefront as a key design variable governing shear-alignment efficiency and network formation. Specifically, by employing two types of h-BN platelets with different aspect ratios and applying a sequential roll-gap control two-roll milling process, optimized in our previous work to maximize alignment, we systematically elucidate how aspect ratio affects reorientation dynamics and structural continuity. Through this framework, we propose a scalable process structure design strategy based on geometry-driven alignment control.

## 2. Materials and Methods

### 2.1. Materials

Polydimethylsiloxane (Sylgard 184 silicone elastomer base, Dow Corning, Midland, MI, USA) was used as the polymer matrix. High-aspect-ratio hexagonal boron nitride (CFP0075, 3M, Saint Paul, MN, USA) was employed as a thermally conductive filler and is denoted as L-BN in this study. Low-aspect-ratio hexagonal boron nitride (CFPS1SF, 3M, Saint Paul, MN, USA) was employed as a thermally conductive filler and is denoted as S-BN.

### 2.2. Fabrication of PDMS/BN Composites

BN composites were fabricated using a PDMS matrix and S-BN and L-BN fillers following the procedure described below. First, PDMS was mixed with a curing agent at a mass ratio of 10:1, after which BN was added and pre-mixed using a paste mixer at 2000 rpm for 5 min. For all composites, BN was incorporated at a loading of 175 wt% relative to PDMS (e.g., 17.5 g of BN for 10 g of PDMS). To achieve uniform dispersion of BN within the PDMS matrix, the pre-mixed paste was subsequently processed through a four-pass three-roll milling procedure.

Random composites were prepared to preserve the random orientation of BN fillers by partially curing the pre-mixed paste in an oven at 80 °C for 10 min, followed by full curing using a hot press with a 200 μm mold at 80 °C and 5 MPa for 1 h. The resulting random composites fabricated with S-BN and L-BN were denoted as s-random and L-random, respectively.

Aligned composites were fabricated using a two-roll milling process, in which the rollers rotated in opposite directions at 120/60 rpm. The roll gap was initially set to 10 μm and was progressively increased in 10 μm increments until a final thickness of 200 μm was reached, while the paste was sequentially fed through the rollers. Both S-BN and L-BN were aligned under identical processing conditions, and the resulting composites were denoted as s-aligned and L-aligned, respectively.

### 2.3. Characterization

Morphological observations were carried out via SEM at an acceleration voltage of 1.5 kV. The crystalline structure and orientation, specifically the (002) and (100) diffraction planes of BN, were investigated using X-ray diffraction (XRD, D2 Phaser, Bruker AXS, Madison, WI, USA). The XRD analysis was performed with Cu Kα radiation at operating conditions of 30 kV and 10 mA, scanning a range from 20° to 80° at a step rate of 0.02°/s. To evaluate the mechanical properties, including Young’s modulus and elongation at break, a universal testing machine (UTM, DRTECH Inc., Seongnam-si, Gyeonggi-do, Republic of Koera) was utilized. In accordance with the ASTM D638-22 standard [38], specimens with a 200 μm thickness were prepared. For each composite category, five independent samples were tested to ensure reproducibility. The thermal conductivity was determined via the transient plane source technique, employing a TPS 2500S system (Hot Disk, Goteborg, Sweden). Measurements were conducted on disk-shaped specimens (3 cm in diameter and 0.2 mm in thickness), and the average results from five consecutive trials were recorded. Furthermore, an infrared thermal imaging camera (M11W, HIKMICRO, Hangzhou, China) was used to monitor the time-dependent surface temperature profiles of the composites.

## 3. Results and Discussion

### 3.1. Aspect Ratio Analysis

To understand the shear-alignment behavior from a geometric perspective, the aspect ratio (AR) of the BN platelets was first quantified. In this study, the aspect ratio was defined as the ratio of the average lateral size to the average thickness of the platelet fillers (AR = lateral size/thickness). To characterize AR, the lateral size and thickness of 50 randomly selected platelets were measured using SEM and AFM, respectively, and the aspect ratio was calculated based on the averaged values.

**Figure 2:**
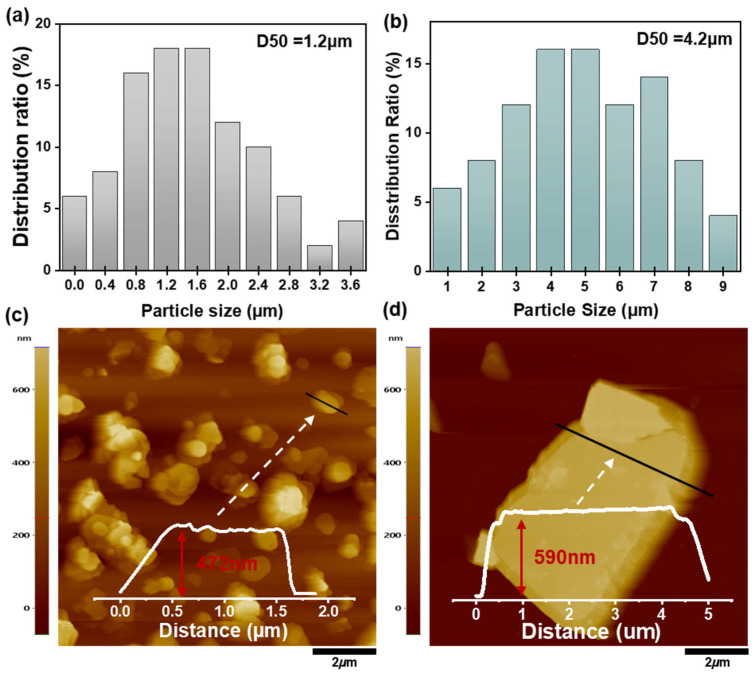
Particle size distribution and AFM thickness of S-BN and L-BN: (**a**,**b**) Size histograms of S-BN and L-BN. (**c**,**d**) AFM height images with line profiles for S-BN and L-BN thickness.

SEM analysis showed that the average lateral size of S-BN (Figure 2a) was approximately 1.2 μm, whereas L-BN (Figure 2b) exhibited a much larger value of ~4.2 μm. Meanwhile, AFM thickness profiles (Figure 2c,d) indicated that the mean thickness of L-BN (590 nm) increased compared with that of S-BN (470 nm), but the increase was marginal relative to the substantial expansion in lateral size. As a result, the calculated aspect ratio of L-BN was 7.12, which is significantly higher than the value of 2.54 for S-BN. Notably, reported AR values for platelet-type h-BN fillers can vary widely depending on the source/grade and the evaluation protocol, and our measured AR values fall within the range commonly reported for commercially available platelet fillers [39]. This AR contrast (S-BN/L-BN) is therefore expected to influence reorientation stability under shear flow and the resulting network continuity under otherwise identical processing conditions, which will be discussed in subsequent sections.

### 3.2. Morphology Analysis

**Figure 3:**
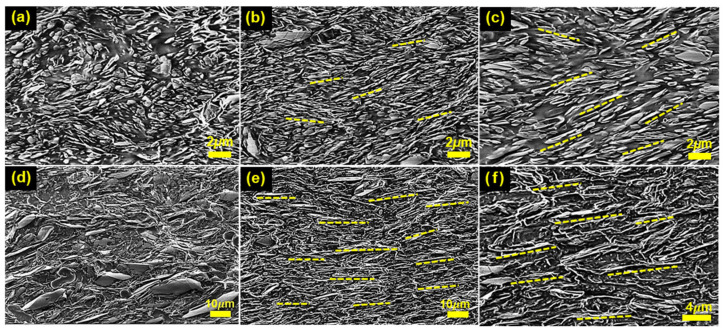
SEM images of PDMS/BN composites: (**a**) S-random; (**b**) S-aligned; (**c**) high-magnification image of S-aligned; (**d**) L-random; (**e**) L-aligned; (**f**) high-magnification image of L-aligned.

To examine how aspect-ratio-dependent shear-induced alignment is reflected in the composite microstructure, cross-sectional images of the PDMS/BN composites were observed by SEM. Figure 3 compares the effects of processing conditions (random/aligned) and filler aspect ratio on the cross-sectional morphology of the PDMS/BN composites. In the random composites (Figure 3a, S-random; Figure 3d, L-random), BN platelets are randomly dispersed within the matrix without any preferred orientation, regardless of their aspect ratio, and polymer-rich gaps are frequently observed at filler–filler junctions. These discontinuous contacts and matrix-dominated regions act as thermal barriers that disrupt heat conduction pathways, thereby hindering efficient heat transport throughout the composite.

In contrast, the composites fabricated by two-roll milling (Figure 3b,c,e,f) exhibit pronounced structural anisotropy induced by shear-driven alignment, and the degree of alignment strongly depends on the filler aspect ratio. As shown in Figure 3b and the magnified image in Figure 3c, the S-aligned composite displays imperfect alignment, with platelets exhibiting noticeable tilting angles relative to the flow direction. This behavior is consistent with the tendency of low-aspect-ratio platelets to undergo periodic tilting/tumbling under shear flow, resulting in frequent deterioration of pathway continuity. By comparison, the L-aligned composite in Figure 3e shows a densely stacked, laminar-like structure with platelets oriented parallel to the flow direction, and the magnified image in Figure 3f confirms a substantially increased overlap contact area between adjacent platelets. Consequently, the aligned L-BN platelets form efficient and continuous thermal conduction pathways, which provide the essential basis for the pronounced enhancement in thermal conductivity.

### 3.3. Crystallographic Analysis of Shear-Induced BN Alignment

**Figure 4:**
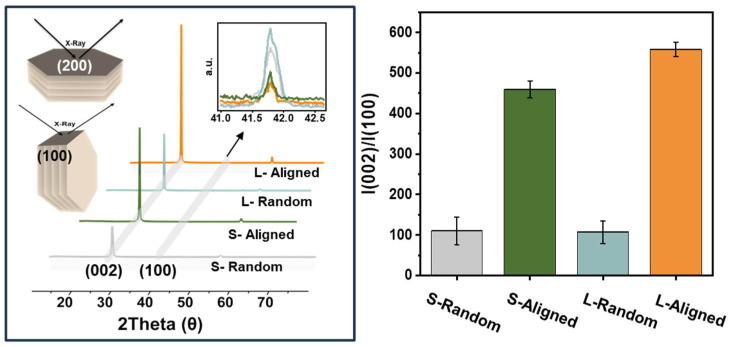
XRD analysis of 175 wt% PDMS/BN composites: XRD patterns; intensity ratio of the (002) to (100) reflections, *I*_002_/*I*_100_.

To quantitatively validate the shear-induced alignment observed in the SEM cross-sections, X-ray diffraction (XRD) analysis was performed. Because platelet-type h-BN has a layered crystal structure, its diffraction intensities are sensitive to particle orientation; thus, changes in preferred orientation can be evaluated by monitoring the relative intensities of characteristic reflections. Figure 4a shows the XRD patterns of the PDMS/BN composites. The diffraction peak at 26.6° corresponds to the (002) basal plane of h-BN, and its intensity increases as a larger fraction of basal planes become preferentially aligned parallel to the sample surface [40]. In contrast, the peak at 41.7° is assigned to the (100) plane, and variations in its intensity relative to (002) can be interpreted as reflecting the extent of basal-plane alignment. The (004) reflection observed at a higher angle is identified as a higher-order diffraction of the (002) plane [41].

For the random composites, the (002) contribution is relatively weak and shows only limited variation among samples, indicating that BN platelets are dispersed without a pronounced preferred orientation. By contrast, the shear-aligned composites processed by two-roll milling exhibit a marked enhancement of the (002) component, confirming that shear processing promotes basal-plane alignment and increases the overall degree of orientation. Figure 4b provides a quantitative comparison of alignment using the intensity ratio *I*_002_/*I*_100_ as an orientation metric. The *I*_002_/*I*_100_ value increases from 459.3 for the S-aligned composite to 558.4 for the L-aligned composite (a ~21.6% increase), with the L-aligned sample exhibiting the highest ratio among all specimens. This trend is consistent with the SEM observations that alignment efficiency is strongly governed by filler aspect ratio. In line with the geometric moment-arm concept introduced in Figure 1, a larger effective platelet radius r (moment arm) can yield a larger shear-driven rotational driving moment under identical processing conditions [42], thereby facilitating reorientation and stabilizing a flow-parallel configuration [43]. Collectively, the increased I_002_/I_100_ ratio in the L-aligned composite quantitatively indicates a higher degree of alignment, implying the formation of a microstructure more favorable for constructing continuous heat-conduction networks.

### 3.4. Mechanical Properties

**Figure 5:**
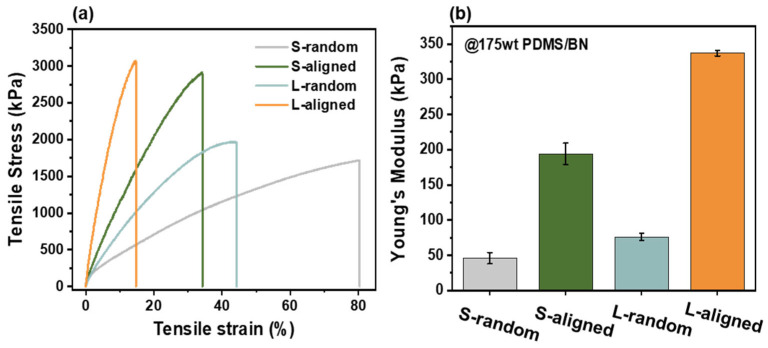
(**a**) Stress–strain curves and (**b**) Young’s modulus of 175 wt% PDMS/BN composites.

The alignment of BN fillers significantly influences not only the thermal conductivity but also the mechanical strength of the composites. To investigate this reinforcement effect, the mechanical performance of the PDMS/BN composites was evaluated. Figure 5a presents the representative stress–strain curves, and Figure 5b shows the corresponding Young’s modulus values.

In terms of stiffness and strength, the results indicate that shear-induced alignment and increased filler aspect ratio substantially enhance the mechanical properties. As shown in Figure 5b, the Young’s modulus increased by approximately 4.2-fold for S-aligned (194 kPa) compared to S-random (46 kPa), and by 4.4-fold for L-aligned (337 kPa) compared to L-random (76 kPa). Notably, the L-aligned composite achieved the highest modulus of 337 kPa, significantly outperforming the S-aligned composite. Similarly, the tensile strength peaked at 3100 kPa for the L-aligned composite, representing a 1.8-fold increase over the S-random (1703 kPa) composite.

This reinforcement is attributed to two synergistic mechanisms. First, the alignment effect enables BN platelets aligned parallel to the stretching direction to effectively bear the applied load and resist matrix deformation. Second, the aspect-ratio-driven stress transfer effect arises from the large modulus mismatch between rigid BN and the soft PDMS matrix, which promotes stress transfer to the fillers [44]. According to shear-lag theory, high-aspect-ratio L-BN provides a larger interfacial contact area, thereby maximizing the efficiency of stress transfer [45,46]. Notably, the L-aligned composite exhibits the highest degree of filler alignment, allowing the high-aspect-ratio L-BN network to act more effectively as a reinforcing skeleton and thereby maximize the overall stiffness of the composite. Conversely, the elongation at break exhibits a trade-off relationship with stiffness [44]. The elongation decreased significantly from 80.28% for S-random to 14.74% for L-aligned. This reduction is due to the confinement effect, where the highly aligned, large fillers restrict the mobility and conformational rearrangement of the polymer chains. Although ductility was reduced, the L-aligned composite retains sufficient flexibility while offering superior mechanical strength, demonstrating its structural reliability for thermal management applications.

### 3.5. Thermal Conductivity and Alignment Effects

**Figure 6:**
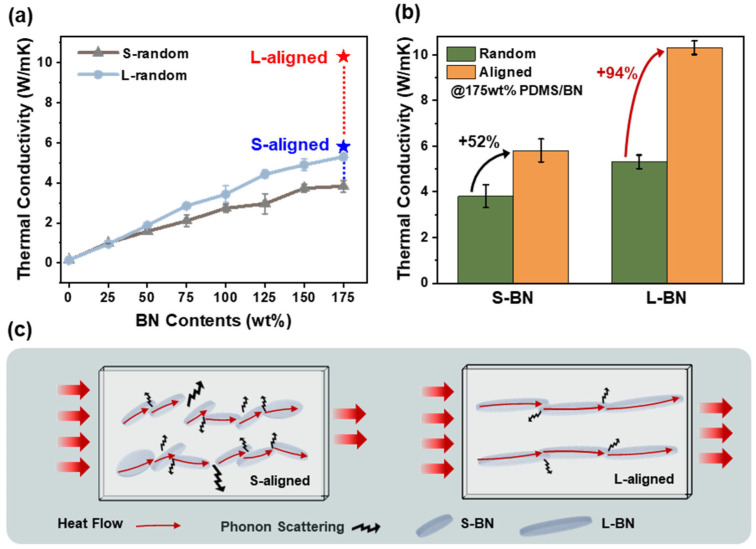
Thermal conductivity and heat-transport schematic of PDMS/BN composites: (**a**) Thermal conductivity of random PDMS/BN composites as a function of BN content (0–175 wt%), together with the 175 wt% aligned composites (S-aligned and L-aligned). (**b**) Thermal conductivity comparison of 175 wt% PDMS/BN composites in random and aligned states (S-BN and L-BN). (**c**) Schematic illustration of heat flow and phonon transport pathways in random versus high-alignment composites.

Figure 6a presents the variation in thermal conductivity of the composites as a function of BN filler loading, ranging from 0 to 175 wt%. In the initial low-loading region (~25 wt%), both S-BN and L-BN composites exhibit similar thermal conductivities, indicating that a continuous thermal percolation network has not yet formed due to insufficient filler-to-filler contacts. However, as the filler loading exceeds 50 wt%, a distinct disparity in thermal conductivity between the two composites becomes increasingly apparent. Across the entire loading range, composites containing high-aspect-ratio L-BN fillers consistently exhibit higher thermal conductivities than the S-BN composites. This behavior is attributed to the ability of L-BN fillers to form longer and more continuous heat conduction pathways within the matrix, thereby facilitating efficient phonon transport. To identify the optimal filler loading, the thermal conductivity of random PDMS/h-BN composites was first screened over a wide range of BN contents (25–175 wt%). Based on this screening, 175 wt% exhibited the highest baseline thermal conductivity and was therefore selected for the subsequent shear-alignment process; higher loadings were not feasible due to a sharp increase in viscosity and loss of flowability during processing. At a high filler loading of 175 wt%, applying shear alignment further amplifies the disparity between the two composites (star markers in Figure 6a). While the S-BN composites show only a limited increase in thermal conductivity after alignment, the L-BN composites exhibit a pronounced enhancement. Specifically, the thermal conductivity of the L-BN composite increases from 5.3 W m^−1^ K^−1^ in the random state to 10.3 W m^−1^ K^−1^ after shear-induced alignment. In contrast, the S-BN composite shows a more modest increase, rising from 3.8 W m^−1^ K^−1^ to 5.8 W m^−1^ K^−1^ upon alignment. This discrepancy in the alignment effect is quantitatively summarized in Figure 6b. The L-BN composites record an increase of approximately 94%, whereas the S-BN composites show a more modest increase of about 52%, rising from 3.8 W m^−1^ K^−1^ to 5.8 W m^−1^ K^−1^ upon alignment. This discrepancy in the alignment effect is quantitatively summarized in Figure 6b. These results demonstrate that a high aspect ratio markedly improves the alignment response under external shear, serving as a critical factor in determining the final thermal performance. The relatively modest thermal conductivity enhancement of the S-BN composites, even after shear-induced alignment, can be rationalized by the schematic illustration in Figure 6c. Heat transport in these composites can be constrained by cumulative thermal resistances along the conduction pathway, including the BN–PDMS interfacial (Kapitza) resistance and the thermal resistance at filler–filler contacts/junctions [15]. For S-BN, fully flow-parallel alignment may not be achieved even after shear processing, which can reduce the effective filler–filler contact area and increase PDMS-mediated interparticle gaps, thereby increasing the frequency with which heat must traverse polymer-dominated boundaries [47]. This can elevate the cumulative resistance and associated phonon scattering, limiting heat-transfer efficiency. In addition, the smaller size of S-BN platelets leads to a larger particle number at the same weight loading, resulting in a higher density of junction points that can further amplify these resistive losses. Moreover, the lower aspect ratio of S-BN may reduce the effective rotational torque under shear, leaving a residual tilted orientation rather than a fully flow-aligned configuration.

When fillers are tilted relative to the dominant heat-transport direction, the effective projected conduction length decreases, forcing heat to traverse more polymer-rich junctions and increasing interfacial thermal resistance. As a result, heat cannot propagate along a continuous filler pathway and must repeatedly traverse relatively low-conductivity matrix interfaces, thereby amplifying the contribution of interfacial thermal resistance to the overall heat transfer process. Consequently, the S-BN network suffers from severe interfacial thermal resistance due to the combined effects of high junction density and short effective conduction length. The accumulation of these resistance components leads to an inefficient S-BN thermal network characterized by high tortuosity, forcing heat to follow zig-zag pathways and hindering linear transport. In contrast, the L-aligned structure shown in Figure 6c demonstrates that high-aspect-ratio L-BN fillers effectively overlap and interconnect along the longitudinal direction, establishing a continuous and linear heat conduction network. This structural characteristic minimizes phonon scattering and matrix-dominated regions, providing the fundamental basis for the significantly superior thermal performance of L-BN composites compared to their S-BN counterparts.

### 3.6. Heat Transfer Platform

**Figure 7:**
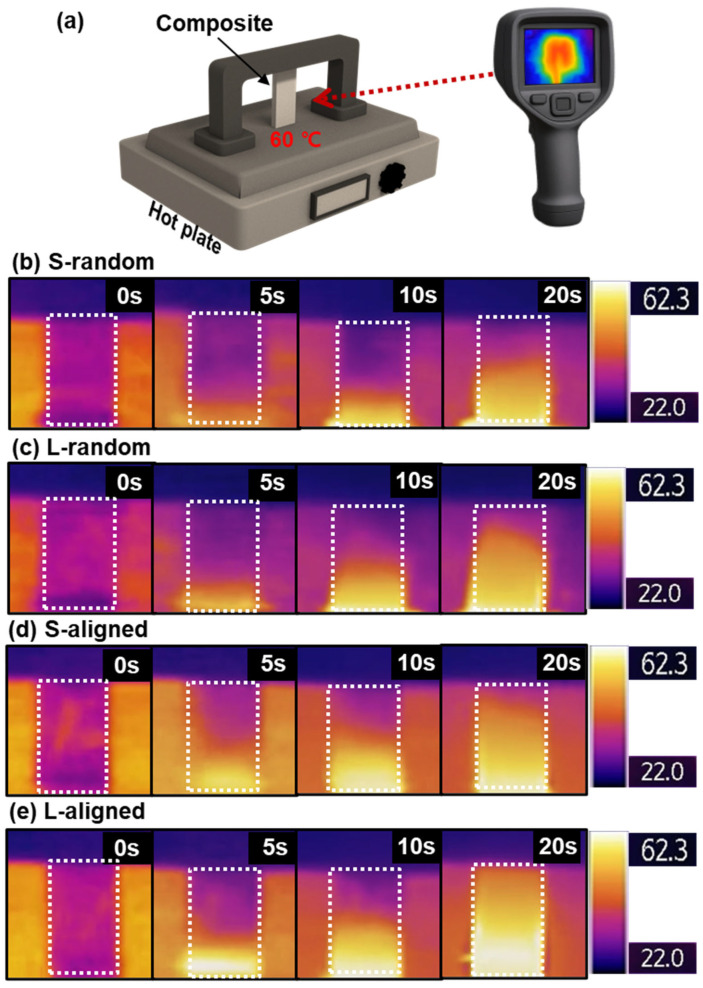
Heat dissipation performance of 175 wt% PDMS/BN composites evaluated by an IR-thermography platform: (**a**) Schematic illustration of the heat dissipation (thermal diffusion) measurement setup. Infrared thermal images of 175 wt% PDMS/BN composites: (**b**) S-random, (**c**) L-random, (**d**) S-aligned, and (**e**) L-aligned, recorded at 0, 5, 10, and 20 s.

To evaluate the practical thermal diffusion behavior of the PDMS/BN composites, a heat dissipation performance test was conducted. As schematically illustrated in Figure 7a, rectangular specimens with dimensions of 1 × 2.5 cm were placed on a hot plate maintained at 60 °C, and the temporal evolution of the surface temperature distribution was monitored using an infrared (IR) camera [48]. Figure 7b–e present the IR thermal images comparing the surface temperature evolution of S-random, L-random, S-aligned, and L-aligned PDMS/BN composites during the heating period from 0 to 20 s.

At 20 s of heating, the L-aligned composite exhibits the fastest and most uniform temperature increase, indicating the most efficient thermal diffusion behavior. The S-aligned composite also shows an enhanced thermal diffusion response compared to the random composites; however, its improvement remains less pronounced than that of the L-aligned composite. In contrast, although the L-random composite demonstrates slightly improved thermal diffusion relative to the S-random composite, it still exhibits localized heat retention and overall inefficient heat transfer when compared with the aligned counterparts. These differences in thermal diffusion behavior can be attributed to variations in the thermal conduction network formed as a function of filler aspect ratio and alignment state. High-aspect-ratio L-BN fillers respond effectively to shear during processing and form a more continuous and better-connected thermal conduction network.

As a result, the overall thermal resistance is reduced, and heat spreads more rapidly within the composite, leading to a faster and more uniform surface temperature rise while minimizing matrix-dominated regions and interfacial thermal resistance. Conversely, S-BN fillers with a lower aspect ratio exhibit reduced alignment efficiency, resulting in a less continuous network and a greater contribution of interfacial resistance, which in turn limits thermal diffusion performance. Overall, the heat dissipation results demonstrate that effective alignment of high-aspect-ratio fillers improves not only intrinsic thermal conductivity but also device-relevant heat dissipation behavior.

## 4. Conclusions

In this study, we delineated a geometry-informed process–structure–property relationship for PDMS/h-BN composites, indicating that platelet aspect ratio plays a central role in shear-induced alignment under two-roll milling. Compared with low-aspect-ratio platelets, L-BN platelets more readily attain and maintain flow-parallel orientation, consistent with improved shear-driven reorientation dynamics. The resulting laminar-like microstructure reduces polymer-rich junctions and enhances platelet connectivity, which lowers interfacial thermal resistance and benefits heat transport. Consequently, the aligned L-BN composite exhibits a thermal conductivity of 10.3 W m^−1^ K^−1^ at 175 wt% loading, exceeding the S-BN counterpart (5.8 W m^−1^ K^−1^) while also achieving the highest stiffness (337 MPa). Taken together, this work underscores that high alignment performance requires coupling process control with geometry-based filler selection, offering a scalable guideline for designing high-performance insulating TIMs.

## Data Availability

The original contributions presented in this study are included in the article. Further inquiries can be directed to the corresponding author.
